# Midwifery and Diseases of Women

**Published:** 1851-03

**Authors:** 


### ARTICLE VI.

### *Midwifery and Diseases of Women.*

*Hemorrhage, Uterine.*—Mr. J. Griffith brings again into notice the great value of oil of turpentine in uterine hemorrhage. He says the most convenient way of giving it is ℥j of turpentine to ℥ss of oil of sweet almonds as a draught, and repeated in five minutes if the symptoms are urgent. (p. 270.)

*Lactation.*—In cases of child-birth where the appearance of milk is delayed, a decoction is made by boiling well a handful of the white Bofareira (Ricinis Communis of botanists) in six or eight pints of spring water. The breasts are bathed in this decoction for fifteen or twenty minutes. Part of the boiled leaves are then spread thinly over the breasts, and allowed to remain until all moisture has been removed from them by evaporation, and probably, in some measure, by absorption. This operation is repeated at short intervals, until the milk flows upon suction by the child, which it usually does in the course of a few hours. If the milk is required to be produced in the breasts of women who have not given birth to or suckled a child for years, the mode of treatment is as follows:—A similar decoction is made, and it is poured while yet boiling, into a large vessel, over which the woman sits so as to receive the vapor over her thighs and generative organs, cloths being carefully tucked around her, so as to prevent the escape of the steam. In this position she remains for ten or twelve minutes, or until the decoction cooling a little, she is enabled to bathe the parts with it, which she does for fifteen or twenty minutes more. The breasts are then similarly bathed and gently rubbed with the hands, and the leaves are afterwards applied to them in the manner described. These operations are repeated three times during the first day, on the second the process is repeated as to the breasts three or four times, on the third day the whole process is repeated. A child is now put to the nipple, and, in the majority of instances, it finds an abundant supply of milk. In the event of success not following, the treatment is continued for another day; and if there be still want of success, the case is abandoned. (Dr. J. O. M'William, p. 316.)

*Oxytoxic.*----The administration of the Indian hemp seems markedly and directly to increase parturient action, and Dr. Churchill states, that it possesses powers similar to those of the ergot of rye in arresting hemorrhage, when dependent upon congested states of the impregnated uterus. [Prof. Simpson, p. 316.]

*Placenta Prævia.*----If the os uteri is not dilated plug the vagina accurately, to produce a coagulum and stay the flooding, to save the child, to prevent the powers of the mother being exhausted by loss of blood, and to favor the dilation of the os. As soon as the state of the os allows, introduce the hand immediately, separate sufficient of the attachment of the placenta to the uterus to allow the hand to pass to turn and deliver. We believe that where the cessation of hemorrhage takes place from the separation of the placental attachment, the placenta being still left in situ, as advocated by Professor Simpson, it is owing to the placenta forming a plug upon the uterine orifices. For the arguments in the defence of the use of the plug and the explanation of its action, see p. 268. [Mr. W. Braithwaite.]

*Sponge Tents.*—An improvement upon Dr. Simpson's method of making sponge tents has been introduced, which consists mainly in not steeping the sponge in a solution of gum; and the advantages of this method are, that "they are made with much greater facility, their being thus compressed into a smaller compass, and that the expansion of the tents is equally gradual, never requiring the aid of tepid water injections, the moisture of the surrounding parts being quite sufficient." (Mr. C. Coates, p. 306.)

*Sterility.*—Dr. E. Williams has described a Japanese remedy for sterility. The tree is one of the order of the Ternstromaceæ of Jessieu, with leaves somewhat larger than those of the congou tea, emitting an odour when bruised resembling pulegium and sabina. The mode of preparation is to take a quantity of the leaves, macerate them in as much rice spirit as will just moisten them, for six hours; then express, and give about a teaspoonful every hour, and two or three doses will invariably bring on the menstrual secretion, which can be maintained by a dose or two daily, for any length of time. (p. 320.)

*Uterine Neuralgia.*----Apply blisters to the hypogastric region, cauterization of the cervix, narcotic injections, with absolute rest and general treatment. (M. Valleix, p. 307.)

*Warty Excrescences.*----Mr. T. W. Nunn, acting on the principle of Dr. Arnott, of using congelation as an anæsthetic agent, has recently applied little wedged-shaped pieces of ice to the necks of large warty growths depending from the labia minora, till they became blanched and cold; and he then removed a great many of them with a single stroke of the knife, without causing the patient any but very slight pain. [p. 814.] *Braithwaite's Retrospect.*

